# Implementing Internet-Delivered Cognitive Behavioral Therapy for Depression and Anxiety in Adults: Systematic Review

**DOI:** 10.2196/47927

**Published:** 2025-01-28

**Authors:** Daniel Duffy, Derek Richards, Garrett Hisler, Ladislav Timulak

**Affiliations:** 1 Amwell Science, Amwell Boston, MA United States; 2 Trinity College Dublin Dublin Ireland

**Keywords:** mixed methods systematic review, internet-delivered cognitive behavioral therapy, iCBT, implementation science, implementation research, depression, anxiety

## Abstract

**Background:**

Scientific implementation findings relevant to the implementation of internet-delivered cognitive behavioral therapy (iCBT) for depression and anxiety in adults remain sparse and scattered across different sources of published information. Identifying evidence-based factors that influence the implementation of iCBT is key to successfully using iCBT in real-world clinical settings.

**Objective:**

This systematic review evaluated the following: (1) aspects that research articles postulate as important for the implementation of iCBT and (2) aspects relevant to the day-to-day running of iCBT services. A mixed methods systematic review using a convergent synthesis design was conducted to bring together evidence across this sparse literature consisting of divergent scientific article types to investigate the implementation of iCBT for depression and anxiety in adults.

**Methods:**

We searched the PsycINFO, PsycArticles, MEDLINE, CINAHL Complete, and Embase databases for any published peer-reviewed scientific articles that report on the implementation of iCBT for depression or anxiety disorders in adults. A total of 40 articles spanning the case study, commentary, meta-analysis, mixed methods study, pilot randomized controlled trial, randomized controlled trial, qualitative study, quantitative study, review, and systematic review article types were identified as eligible for this mixed methods review. Data were analyzed qualitatively using the descriptive-interpretive approach.

**Results:**

The first domain highlighted the impact of therapist and patient attitudes when implementing iCBT, the superiority of guided iCBT over unguided iCBT, its noninferiority to equivalent face-to-face treatments, and its utility outside of the original target of mild-to-moderate depression and anxiety. In total, 3 subdomains were identified under the second domain: (1) the management of iCBT in the workplace, detailing the importance of managing the iCBT service, related staff, and their motivations for using it; (2) the practice of iCBT in the workplace, describing the therapeutic aspects of iCBT provision, such as the provision of support, the background of supporters, and screening procedures; and (3) contextual considerations, detailing the impact of governmental legislation on therapy conducted over the internet, the lack of an iCBT workforce as a limiting factor, and the cost estimates associated with iCBT provision.

**Conclusions:**

Broadly, the findings describe several aspects that should be taken into account when researchers or practitioners implement iCBT as part of their work. However, the findings should be interpreted with caution, as the articles reviewed spanned many article types, and few of the included studies were directly focused on evaluating the implementation of iCBT. While findings provide insight into important factors to consider during iCBT implementation, these findings and their limitations highlight the need for more implementation-specific research in this area.

## Introduction

### Background

Internet-delivered cognitive behavioral therapy (iCBT) for depression and anxiety has been developed to help increase access to evidence-based therapies. There is empirical support for its use in treating depression and anxiety [1-4]. End users experience it positively [5] and find it to be satisfactory and acceptable [1,6,7]. However, disseminating iCBT at scale remains a challenge [8,9], and COVID-19 has brought its relevance to light now more so than ever [10-12]. A 2019 commentary [13] discussed the evidence-to-practice gap in digital mental health treatments. The authors postulated that the reason for this gap is a lack of knowledge in the field of iCBT around implementing interventions within routine care. They suggested the adoption of implementation science methodologies to bridge this evidence-to-practice gap.

Implementation science has been defined as the scientific study of methods to promote the systematic uptake of research findings and other evidence-based practices into routine practice to improve the quality and effectiveness of health services and care [14]. Central to this definition is the problem statement behind it: it takes almost 17 years for health care research to achieve its intended benefit, which is termed as the “evidence-to-practice gap” [15,16]. As a newly emerging academic field, implementation science is largely integrative; it borrows and adapts theories from multiple fields and uses these to understand the determinant mechanisms as to why (or why not) a specific implementation succeeds [17]. Implementation science theories provide a framework that allows for implementation plans to be developed and relevant outcomes to be measured [18], and it has been posited that using these methodologies in future studies of iCBT could generate learnings relevant to its real-world application [13,19,20].

In a recent review focusing on determinants of implementation for eHealth interventions [8], 37 determinants associated with successful implementation were identified. However, it should be noted that “eHealth interventions” in this case contained a wide variety of digitally enabled interventions, including iCBT and psychotherapy delivered via videoconferencing. When comparing iCBT and other eHealth interventions, “complexity” is a factor for consideration, that is, the degree to which an intervention contains multiple components that require interaction from many individuals, from various levels within an organization, to enact the intervention effectively [21]. iCBT’s level of complexity is highlighted in service illustration articles by Titov et al [22,23]; for example, therapists’ skill set to operate iCBT efficiently (technical knowledge and constructing written messages), revised services delivery pathways, adherence to regulatory frameworks, and newly aligned clinical governance procedures are some elements of how delivering iCBT may differ from more traditional or less complex services. Conversely, although administering psychological therapy through videoconferencing software may require some altering of specific therapeutic skills and technical upskilling [24], relative complexity across other areas may be lower (eg, referral pathways and wider system integration). Similarly, some authors have illustrated the need for both iCBT [25,26] and telehealth-specific competency frameworks [27], further illustrating the need for specialized skills to extend the traditional therapist skill set.

Attempts to mobilize implementation science information on eHealth interventions, generally to the point of having pragmatic, clinical relevance for iCBT, have been sparse [28]. As a consequence, the availability of implementation findings relevant to iCBT remains low. This study is a mixed methods systematic review that aimed to account for literature that specifically references or can inform factors relevant to the implementation of iCBT, specifically for depression and anxiety treatment in adults. The benefit of a mixed methods systematic review over traditional systematic literature reviewing is that it seeks to extract relevant information across qualitative, quantitative, review, and illustration-based articles. Mixed methods synthesis affords a way to effectively capture this information and synthesize it qualitatively to produce relevant insights into the implementation of iCBT. Therefore, a convergent integrated approach to the mixed methods review was chosen due to its appropriateness over other review methods for the subject; a traditional systematic review on the implementation of iCBT for depression and anxiety would not be appropriate due to insufficient qualitative or quantitative findings to generate insights [29]. Furthermore, there are no restrictions imposed on the type of evidence included within the synthesis, which aligns with the anticipated variety of articles that would be identified [30]. The disorder domains of depression and anxiety were chosen due to them being the most substantive areas of research for iCBT.

A mixed methods systematic review departs from and complements previous work in the ways mentioned subsequently [8]. First, it will specifically focus on iCBT-based interventions, which can be considered relatively “complex” in their administration [21]. Second, it will provide a rich description of the current “practice behind the science” by focusing on reportage within the methods, results, and discussion sections of the articles. Third, it will contribute to the existing literature regarding specific implementation strategies that are associated with the use of iCBT [31]. Finally, it will allow the interpretation of research findings in a way that is hoped to be productive for future implementations specific to iCBT for the treatment of depression and anxiety.

### Review Objectives

The overarching objective of the review was centered on the pragmatic question of “What can we learn from published peer-reviewed literature about the implementation of iCBT for depression and anxiety?” This objective was further broken down into 2 domains of focus, on which data extraction and subsequent data analysis were based (refer to the Methods section).

The first domain was centered on *implementation insights derived from iCBT research*. This objective and domain center on understanding the novel information that is often presented in published research and how this information can have relevance and be mobilized for the benefit of iCBT implementation.

The second domain was *implementation process considerations for the successful implementation of iCBT*, which consisted of establishing the strategies that are used within the literature to facilitate the implementation of iCBT. According to implementation science literature, implementation strategies are methods used to facilitate the implementation of an intervention, where strategies can consist of training packages, management approaches, developing protocols for intervention use, etc [31,32].

## Methods

### Design

Because implementation science information on eHealth interventions that has pragmatic, clinical relevance for iCBT has been sparse and scattered across different article types, a mixed methods systematic review was conducted. This mixed methods systematic review used a convergent integrated approach and was conducted to identify literature that was central to the review objective mentioned earlier [30,33,34]. The convergent integrated approach to conducting a mixed methods systematic review consists of “qualitizing” numerical or statistical findings; that is, quantitative findings are extracted and allocated textual descriptions to allow for integration and simultaneous synthesis with other qualitative data. The resulting qualitative data were then analyzed using the descriptive-interpretive approach [35]. This review was not registered, and a review protocol was not prepared.

### Search Strategy

The general search strategy used was as follows: (“ICBT” OR “CCBT” OR “internet-delivered CBT” OR “internet-delivered cognitive behavioural therapy” OR “internet-delivered cognitive behaviour therapy” OR “internet-based cognitive behaviour therapy” OR “internet-based cognitive behavioural therapy” OR “internet-administered cognitive behaviour therapy” OR “internet-administered cognitive behavioural therapy” OR “internet-supported cognitive behaviour therapy” OR “internet-supported cognitive behavioural therapy”) AND (“Anx*” OR “depress*” OR “low mood” OR “GAD” OR “phobia” OR “SAD”) AND “Implement*.” Databases searched included PsycINFO, PsycArticles, MEDLINE, CINAHL Complete, and Embase. A full description of terms and derivatives for the different databases is included in Multimedia Appendix 1. Search engine limitations required that the search date had to start from 2007. The search was initially conducted in June 2020 (January 1, 2007-June 1, 2020) and further repeated in September 2021 (June 1, 2020-August 31, 2021) to identify any new or relevant publications. The inclusion and exclusion criteria are given in Textbox 1.

**Inclusion and exclusion criteria.**

**Inclusion criteria**

- Study provides reports on outcomes related broadly to the 2 domains of interest:
  Implementation insights derived from internet-delivered cognitive behavioral therapy (iCBT) research
  Implementation process considerations for the successful implementation of iCBT
- The study types included in the review:
  Empirical research, encompassing pre-post experimental (eg, feasibility or randomized controlled trial), case study, observational, or qualitative designs in naturalistic, nonefficacy settings
  Review-type studies, including systematic, meta, umbrella, narrative, and scoping reviews
  Service-illustration articles that report on the effectiveness of iCBT clinics over time or describe their operating model
- Studies targeting adult patient populations, mental health care workers (eg, clinicians, therapists, and service managers), or prospective users of iCBT
- The study must be conducted in reference to iCBT (eg, patients undertaking iCBT, clinicians or therapists, or patients reporting on their views of iCBT).
- The study must be primarily conducted in reference to depression and anxiety disorders (eg, patients undertaking iCBT for depression and anxiety, clinicians and therapists, or patients reporting on their views of iCBT for depression and anxiety)

**Exclusion criteria**

- Nonpeer reviewed research
- Research not in the English language
- Protocols
- Dissertations (due to the difficulty in identifying and accessing these at a wide scale)
- Book chapters
- Conference presentations and abstracts
- Research with participants aged <18 years
- Studies reporting only on clinical effectiveness data with no information on the implementation of iCBT reported on in the study.

### Screening

The screening consisted of two steps: (1) review the title and abstract and (2) review the full-paper. We chose to first review the titles and abstracts of all identified records due to the nature of this review and the wide range of study types that were anticipated to result from the search. For example, it was noted throughout the reviewing process that articles frequently cited the terms “implementation” or “feasibility” in the title but failed to provide any relevant information under these constructs when abstracts were reviewed. Where articles provided inadequate information in their abstracts (eg, “the results inform the feasibility of implementing iCBT within XYZ context”) to apply the inclusion criteria, DR acted as a second reviewer for these abstracts and consulted with DD (primary reviewer) to make a decision on inclusion or exclusion. Once step 1 was completed, full texts of all articles were reviewed by DD at full text to discern their relevance to the domains of interest. During this review, articles were rejected during full text screening for the following two reasons: (1) incorrect record specification from the databases (eg, conference presentations being mislabeled) and (2) provided little (eg, minor comments relating to the implementation of iCBT within “future research” sections) or no information on implementation. Once all articles were screened and the final dataset established, data extraction commenced.

### Data Analysis

#### Meaning Unit Extraction

Meaning unit extraction began by identifying qualitative meaning units within the methodology, results, and discussion sections of articles relevant to the study objective, that is, learning about the implementation of iCBT for depression and anxiety. Meaning units are discrete data chunks (either paragraphs or sentences) that contribute stand-alone meaning toward a particular research question or objective [36]. Throughout the mixed methods systematic review process, relevant quantitative findings were translated (or “qualitized”) to qualitative meaning units. The resulting qualitative meaning units were identified and subsequently extracted to an Excel (Microsoft Corporation) file for analysis and assigned relevant identifiers. In addition to assigning identifiers, each meaning unit was also assigned brief, textual summary labels that provided a way to quickly identify the information being conveyed by longer meaning units.

To address the review objective of learning about the implementation of iCBT for depression and anxiety, 2 main focus areas were identified that allowed for the structuring of the relevant data to guide meaning unit extraction.

The first focus area was the implementation process considerations for the successful implementation of iCBT. It consisted of the following questions: (1) What strategies do articles report on that are related to the process of implementing iCBT (eg, training clinicians or therapists, screening procedures, referral pathways, and service operations)? (2) Do articles report on the impact of these strategies on specific stakeholder groups (eg, patients and clinicians or therapists)? and (3) Do articles acknowledge or cite factors within the context of the implementation (eg, governmental policy, service infrastructure, and funding)?

The second focus area was the implementation insights derived from iCBT research. It involved the following questions: (1) What implications do authors of the included studies cite as important for the future of the implementation of iCBT? and (2) How do authors interpret their findings in discussion sections of articles, and can these interpretations have implications for how iCBT is implemented?

#### Category Generation

Meaning units within these 2 focus areas were then further analyzed for similarities and clustered together into categories and subcategories grouping meaning units of similar meaning. The categories and subcategories were named to capture the meaning of the meaning units they contained. The process of organizing and naming categories and subcategories was an ongoing activity led by DD and involving feedback from DR and another colleague. The consensus was agreed upon through group discussion, where the core component of each subcategory was established (eg, this subcategory describes x), and the revised category names were generated based on this shared understanding. As per the descriptive-interpretive approach, it was important that progress was continuously audited through group meetings with DD, DR, JP, and LT.

## Results

### Overview

**Figure 1:**
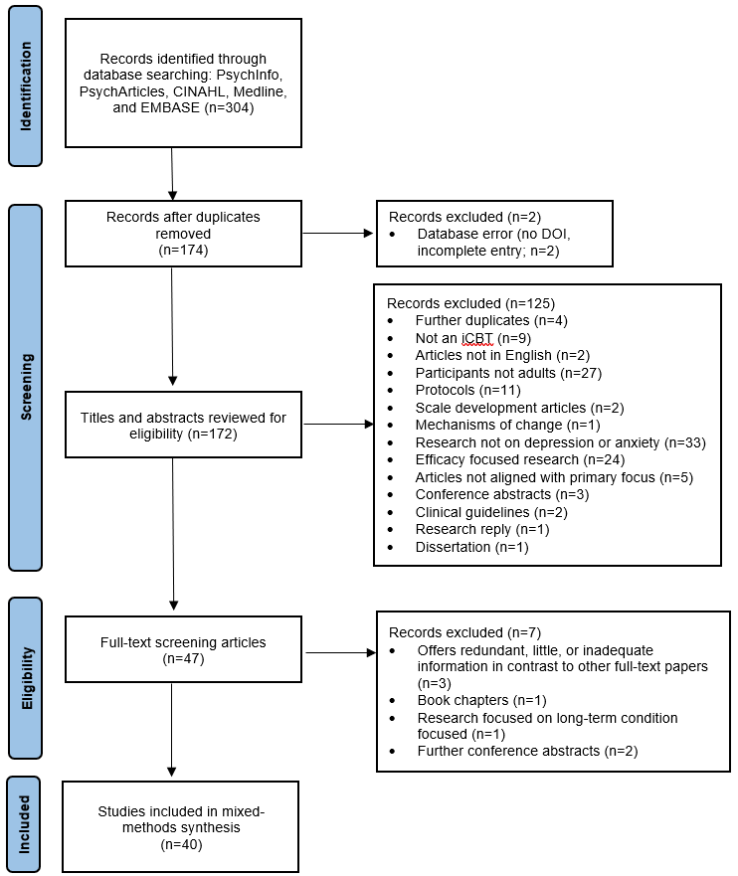
PRISMA (Preferred Reporting Items for Systematic Reviews and Meta-Analyses) diagram for mixed methods systematic review search. DOI: digital object identifier; iCBT: internet-delivered cognitive behavioral therapy.

A total of 40 eligible articles published between 2010 and 2021 were included in the mixed methods synthesis. Multimedia Appendix 2 provides the types of articles included in this mixed methods systematic review and brief summaries of these included articles. Article types included in this review were case study (1/40, 2%), commentary (4/40, 10%), meta-analysis (5/40, 12%), mixed methods study (3/40, 8%), pilot randomized controlled trial (1/40, 2%), randomized controlled trial (2/40, 5%), qualitative study (5/40, 12%), quantitative study (13/40, 32%), review (3/40, 8%), and systematic review (3/40, 8%). Multimedia Appendix 3 [4,9,22,23,28,37-71] includes a numbered list of all articles analyzed as part of this mixed methods systematic review. The PRISMA (Preferred Reporting Items for Systematic Reviews and Meta-Analyses) flow diagram constructed to illustrate the search findings is illustrated in Figure 1 and the PRISMA checklist is provided in Multimedia Appendix 4.

### Domain and Category Structure

Two domains (and lower subdomains, categories, and subcategories) were identified: (1) implementation insights derived from iCBT research and (2) implementation processes related to the successful implementation of iCBT in care settings (Tables 1 and 2). Subcategories are discussed as talking points within each category as opposed to adding further organizational levels within this study.

**Table 1:** Categories and subcategories identified under the domain implementation insights derived from internet-delivered cognitive behavioral therapy (iCBT) research and illustration of the number of contributing articles (N=40).

Category and subcategory		Articles, n (%)	Study reference number
**Clinician attitudes toward iCBT**			
	Negative attitudes toward iCBT can impact referral rates and patient outcome	10 (25)	[4,5,13,23,25-27,29,34,36]
	Positive attitudes toward iCBT can increase acceptability and help to grow iCBT in service	3 (8)	[29,31,34]
**Patient attitudes toward iCBT**			
	Positive attitudes toward iCBT content, support, privacy, and convenience of iCBT can foster engagement	10 (25)	[3,8,27,29,32,33,36,72-74]
	Attitudes as moderators of clinical outcome, perceived helpfulness, and adherence	3 (8)	[18,36,73]
	Negative attitudes relate to preference for face-to-face therapy and issues with the utility of iCBT to patient needs	5 (12)	[25,26,36,72,73]
The delivery of internet-delivered therapies can be helped by technological and clinical augmentation		7 (18)	[11,16,23-25,27,34]
**Specific patient characteristics need to be considered when implementing iCBT**			
	Age is negatively associated with adherence and clinical outcomes in guided iCBT and not associated with symptom deterioration in unguided iCBT	4 (10)	[1,20,36,74]
	The relationship between gender or sex and adherence is unclear in iCBT overall, but gender or sex is not associated with symptom deterioration in unguided iCBT	2 (5)	[1,36]
	Patient technological literacy is tentatively positively associated with adherence and clinical outcomes in iCBT	2 (5)	[36,75]
	Medication and alcohol use are not associated with iCBT adherence	1 (2)	[36]
	Racial or ethnic minority group membership is negatively associated with adherence to iCBT	1 (2)	[35]
	The relationship between adherence and marital status, employment status, and education level is mixed overall, but they are not associated with symptom deterioration in unguided iCBT	3 (8)	[1,36,74]
	Having a lower income is positively associated with dropout	1 (2)	[74]
	Comorbidity of disorders can moderate treatment outcome	1 (2)	[74]
	Making sudden clinical gains is associated with greater improvements after the treatment	1 (2)	[24]
	The severity of depression can positively impact clinical outcomes and adherence	2 (5)	[10,36]
	Symptoms of depression can negatively impact iCBT adherence	2 (5)	[12,18]
	Chronic mental health problems are negatively associated with iCBT adherence	1 (2)	[74]
Guided iCBT is superior to unguided iCBT in regard to symptom outcomes and adherence		11 (28)	[1,7,17,23-25,27,34,36,73,74]
iCBT is as effective as face-to-face delivery of the same protocol, yet preference is often for face-to-face treatment		13 (32)	[6,11,13,14,16,23-25,27,29,36,72]
iCBT appears to be effective beyond the original target of mild-to-moderate depression and anxiety		10 (25)	[8,10,13,14,23,26,27,29,32,33]
**Conducting future research that has relevance for iCBT implementation is important**			
	More implementation research is needed to understand the uptake of iCBT within routine care	9 (22)	[7,18,23-25,27,28,33,34]
	More research is needed on adverse events to understand the negative effects of iCBT	2 (5)	[19,23]
	More research is needed to understand the relationship between adherence and iCBT	4 (10)	[8,23,28,36]

**Table 2:** Subdomains, categories, and the contributing articles identified under the domain implementation processes related to the successful implementation of internet-delivered cognitive behavioral therapy (N=40).

Subdomain and category and subcategory				Articles, n (%)		Studies
**Management of iCBT in the workplace**						
	Successful training of supporters is important for the provision of iCBT			8 (20)		[4,14,16,22,26,29,31,34]
	Training stakeholders within the health system is important in creating awareness of iCBT			2 (5)		[14,29]
	Effective management of risk and adverse event management in iCBT is important for its delivery			10 (25)		[5,8,12,14,22,23,27,30,33,75]
	iCBT should be delivered through secure and interoperable systems that facilitate clinician and client access			11 (28)		[4,11,12,15,24,27,29,30,34-36]
	**Operational considerations for managing iCBT and related staff are important**					
		Effective management and leadership support facilitate the implementation	7 (18)		[12,14,22,24,29,31,34]	
		Management of workplace resources is necessary to facilitate staff time to use iCBT	4 (10)		[4,26,29,31]	
		Staff motivation to use iCBT needs to be fostered	4 (10)		[26,27,29,34]	
		Use of routine monitoring of iCBT to convey intervention effectiveness and enhance its delivery	5 (12)		[27,29,33,34,74]	
		Effective marketing and service promotion enhance the uptake of iCBT	6 (15)		[4,18,21,29,33,35]	
		Staff recruitment and retention in iCBT is a challenge that needs to be mitigated against	2 (5)		[4,14]	
		Scaling of iCBT within services is challenging and requires multiple considerations (eg, infrastructure, funding, proper testing, and governance)	7 (18)		[4,12,14,15,22,33,74]	
**The practice of iCBT in the workplace**						
	Appropriate referral pathways and management of waiting times are important for the delivery of iCBT			19 (48)		[2,4,9,12,14,18,20-23,27,30-33,35,73-75]
	Screening and inclusion criteria for accessing iCBT need to be thoroughly defined			21 (52)		[8,9,11-14,16,18,20,22,24,26,29,30,32,33,35,72-75]
	**Considerations of the level of support for patients are crucial in the provision of iCBT**					
		Positive impact of support on patients	11 (28)		[2,3,5,10,13,23-25,33,36,73]	
		The quality of support impacts the success of iCBT provision	13 (32)		[8,12,16,22-24,27,29,30,34,36,73,74]	
		Appropriate considerations should be given to the mediums and modalities of support to fit service and user needs	20 (50)		[2,8,11-13,16,21-27,30,32-35,73,74]	
		The time demand associated with the provision of support needs to fit service and user needs	18 (45)		[2,7,11,12,18,21-24,26,27,30,32-35,73,74]	
	The optimal personal and professional background of the supporter needs to be considered in the provision of iCBT			17 (43)		[4,11,12,14-16,21,23-27,30-33,35]
**Contextual considerations**						
	Governmental and health care regulations affect the implementation of iCBT			10 (25)		[4,12-15,24,29,31,33,34]
	Lack of workforce availability for iCBT as a limiting factor in the provision of iCBT			4 (10)		[5,27,31,34]
	Considering the cost estimates associated with iCBT for patients and health care providers before implementing			11 (28)		[4,8,12-14,22,26,27,29,31,75]

#### Domain 1: Implementation Insights Derived From iCBT Research

This domain includes categories that contribute to the success of iCBT in either research or routine practice settings or further learning to inform it.

##### Category 1: Clinician Attitudes Toward iCBT Affect Patient Outcomes and Implementation of iCBT

Clinician attitudes toward iCBT are mixed [48]. Negative attitudes hinder the successful dissemination of iCBT to clients [58]. These attitudes consist of skepticism about the effectiveness and quality of iCBT [9,38], technological limitations of iCBT [56], the inability to generate a therapeutic alliance through this medium [9], preference for face-to-face therapy [42,65,67], the perceived lower priority of the intervention in the workplace [28], and its highly standardized nature being incompatible with other psychological interventions [28,58]. Such negative attitudes can arise from a lack of iCBT exposure or training [59] and can be transferred to patients and undermine patient outcomes [28]. Accordingly, there is a need to engage with these negative attitudes to successfully implement iCBT [28,65].

In contrast, positive attitudes toward iCBT can increase acceptability and help grow iCBT in service. Positive attitudes include acknowledging the benefits of iCBT in terms of time efficiency, cost-effectiveness, evidence base, program design quality, accessibility, and ability to bridge treatment gaps for those on waiting lists [28]. Professionals with more experience implementing iCBT regard iCBT more positively in terms of its applicability to service provision [62]. Even health care professionals with little exposure to iCBT are generally positive and accepting toward iCBT but have biases around suitability and large knowledge gaps [65].

##### Category 2: Patient Attitudes Toward iCBT Affect Engagement, Adherence, and Outcomes

The first subcategory under this larger category relates to patients reporting positive attitudes toward the iCBT treatment, its content, and the associated therapist support they receive [28,41,63,67,69,71]. They also report strong motivations to seek out iCBT [45] and acknowledge advantages in terms of convenience, cost, privacy, and self-directed nature [38,67-69]. A second subcategory relates to attitudes, where greater positive initial attitudes predict greater symptom reduction and adherence, and improvement or decline in attitudes during treatment predicts better or worse adherence and outcomes [52,67,69]. The third subcategory relates to negative attitudes, which can be a barrier to treatment success [59]. Such negative patient attitudes include skepticism toward the effectiveness and credibility of iCBT [68,69] as well as toward motivation and accountability to progress through iCBT [68]. There is also a reported preference for face-to-face therapies over iCBT [58,67-69]. Interestingly, offering iCBT as a waiting list treatment can create “unfavorable comparisons” between iCBT and face-to-face therapy, resulting in negative perceptions of iCBT [69].

##### Category 3: The Delivery of iCBT can be Augmented by Technological and Clinical Design Factors

iCBT has been augmented with novel design elements or treatment strategies to understand their utility and benefit. Such elements and strategies include integrating sensors [57], gamification [57], transdiagnostic elements [38,57,58], iCBT as an add-on or adjunct to existing care pathways [47,56,65], incorporating “persuasive technology” components [57], iCBT use in a blended care model [50,56,57], and its use as a first-line intervention to promote interest in further mental health care [65].

##### Category 4: Patient Characteristics Related to iCBT Outcomes

A variety of demographic, medical, and technological factors have been implicated to affect iCBT and are reported in the subsequent subcategories. There is mixed evidence for age influencing iCBT outcomes wherein null or negative associations have been observed between age and adherence [67,71] and symptom reduction [39,54]. Mixed results are also reported for gender and sex. Females can have greater or similar iCBT adherence to males [67], and sex was found to be associated with symptom deterioration in unguided iCBT [39]. Perceived technological literacy has been posited to promote adherence [67,70] and clinical outcomes [70], though the evidence is unclear regarding the impact of tech literacy on iCBT outcomes [67]. A review reported that patient-reported medication and alcohol use were not associated with iCBT adherence [67]. Racial and ethnic minority group membership has been negatively associated with adherence to iCBT [66]. Both positive and negative associations have been observed for marital status, employment status [39,67,71], and education level [67] with adherence. Individuals with both lower income levels and marital status of single were more likely to drop out from iCBT [71]. Comorbidity of mental health disorders can reduce the effect of iCBT treatment [45]. Chronic mental health problems are negatively associated with iCBT adherence [71].

Making sudden, large clinical gains on symptom measures at the start of treatment is associated with greater improvements after the treatment [57]. The severity of depression can positively impact clinical outcomes of depression; for example, higher pretreatment severity results in greater effect sizes [4,67]. Symptoms of depression have also been found to negatively impact iCBT adherence, where symptoms like low motivation have been found to be negatively associated with iCBT adherence [23,52]. Chronic mental health problems are further associated with iCBT adherence, where years of living with chronic mental health conditions was found to be negatively associated with adherence to iCBT [71].

##### Category 5: Guided iCBT as Superior to Unguided iCBT in Regards to Symptom Outcomes and Adherence

Guided iCBT shows superiority over unguided iCBT in terms of adherence and clinical outcomes [44,51,56-58,65,67,71]. However, an individual participant data meta-analysis [39] postulates that the small effects achieved by unguided iCBT are superior to control groups (or no intervention) and can be best used when implemented at scale, such as at the public health level. The therapist element of guided iCBT, in particular, is posited to improve adherence to iCBT [65,67]. Guided iCBT support also fulfills an expressed need to navigate through and explain therapeutic content when patients encounter difficulties [69].

##### Category 6: iCBT Is as Effective as Face-to-Face Delivery of the Same Protocol, Yet Preference is Often for Face-to-Face Treatment

iCBT produces similar adherence [38,57] and clinical outcomes [48,50,56,57] to equivalent face-to-face therapy. iCBT has advantages over face-to-face therapy in terms of time efficiency, access rates [28,57], and its ability to deliver standardized treatment [22,47]. However, patients demonstrate a preference for face-to-face treatment over iCBT, which can be a reason for the dropout of iCBT [28,43,58,67,68]. In such instances, these preferences for iCBT over face-to-face treatment can be reduced by introducing a time delay when accessing treatment [43].

##### Category 7: iCBT Appears to be Effective Beyond the Original Target of Mild-to-Moderate Depression and Anxiety

iCBT is not typically offered for severe presentations of depression and anxiety [59], but real-world data illustrate that a large proportion of patients seen by iCBT clinics have symptoms in this range [22]. Patients with severe symptoms at baseline can make large clinical gains [4,38,45,56], show comparable adherence rates to patients with less severe symptoms [38], and, in some cases, produce larger gains than patients with more severe symptoms [38]. Studies requiring greater treatment-seeking behaviors tend to recruit individuals with more severe symptoms of depression, illustrating the willingness and motivation of this cohort to initiate treatment [38,56]. After treatment initiation, the effect of higher pretreatment severity on adherence and completion is unclear, with a study positing that higher pretreatment severity may be associated with lower iCBT completion rates [64]. Similarly, patients whose symptoms are in the subclinical range also benefit from iCBT [56,63]. Those with suicidal ideation are also found to benefit from iCBT [38,48,56]. iCBT may also be applicable to conditions where depression is secondary to the presenting problem (eg, addiction, trauma, schizophrenia, and bipolar disorder [28,38]).

##### Category 8: Future Research on Implementation Critical to Advancing iCBT

Several key areas critical to advancing the implementation of iCBT were apparent. First, a plethora of articles stated a lack of research that details the process of implementing iCBT in naturalistic settings; therefore, more research is needed to understand and improve iCBT uptake [38,44,52,56-58,60,64,65]. Second, more research is needed regarding adverse events within iCBT and that current reporting of the adverse events in studies is poor [53,56]. Third, more research is needed to understand the relationship between adherence and iCBT outcomes [45,56,67]. It was also suggested that the definition of “dropout” should not be conceptualized in a binary way because varying dosages of iCBT have been found to produce positive clinical change when less than the intended program is completed. A further study stated that high rates of dropout observed in iCBT research should similarly be expected for practical implementations [60].

#### Domain 2: Implementation Processes Related to the Successful Implementation of iCBT

This domain includes categories of factors that are important for the successful implementation of iCBT.

##### Subdomain 1: Management of iCBT Day-to-Day Workplace Operations

This subdomain consists of factors important for managing the day-to-day operations of iCBT, with categories pertaining to the training of staff, risk management, marketing and service promotion, IT infrastructure, working with other services, and managing the staff who work in the provision of iCBT.

###### Category 1: Successful Training of Supporters Is Important for the Provision of iCBT

Effective training of supporters in iCBT requires technical training in the use of the program [22,50], developing competencies around computer skills and web-based written communication [9,22,62], and practice providing support to fictional patients [50]. Training on writing skills (as many iCBTs rely on supporters messaging the patients on the iCBT platform) should occur before patient interactions rather than through trial and error during their interactions with patients [9]. Training supports, including a manual [59,65], giving clinicians access to training resources [28,65], and providing them with feedback on their written reviews [50], were considered helpful. A study stated that there are limited opportunities for support training in iCBT [37].

###### Category 2: Educating Stakeholders Within the Health System Is Important to Create Awareness of iCBT

Training and educating other relevant stakeholders (eg, nonclinical staff, referral providers, and patients) about the benefits of iCBT is important to create awareness of the intervention and its clinical effectiveness [28] increasing the perceived viability of the intervention as a treatment option [22].

###### Category 3: Effective Management of Risk and Adverse Events in iCBT Is Important for Its Delivery

Successfully implemented iCBT has to be supported by a risk monitoring system (eg, suicidal risk measures) that alerts clinicians to risk (eg, triggered automated messages within the iCBT software) and allows clinicians to act on indicated risk (eg, clinician contacts identified risk cases; [22,23,37,42,45,56,64,70]).

###### Category 4: iCBT Requires Secure, Interoperable Systems That Facilitate Clinician and Client Access

Reviewed articles suggested that iCBT interventions should be hosted on secure servers [28,38,66], should be optimized to run on a variety of mediums (tablets, desktops, and phones) [38], should be integrated with larger patient databases [9,47,61], and should have security standards that adhere to relevant governing bodies [23,57]. Internet connection difficulty [49,65], enabling service computers to access iCBT and its related websites, a lack of integration of iCBT apps with health care records [65], and providing patients access to technology to use iCBT have been cited as limiting factors and may contribute to patient dropout [49,67].

###### Category 5: Operational Considerations for Managing iCBT and Related Staff Are Important for the Successful Implementation

This category details operational factors that impact the successful implementation of iCBT within clinics or workplaces. First, reviewed articles suggest that management and leadership are important to implement iCBT [28] and include activities such as developing guidelines and service procedures [23,28,37,57], change management [22], and planning implementation and engaging stakeholders [28,65]. In addition, management of workplace resources is necessary to facilitate staff time to use iCBT. For instance, clinicians with already high workloads may experience time shortages for administering or reviewing the program [28,59]. iCBT clinic managers worry about clinicians balancing iCBT and face-to-face work workloads [9], and it may be necessary to have a dedicated workforce to support iCBT delivery [62].

Second, staff motivation to use iCBT needs to be fostered, as motivation to use iCBT is “essential” [59] because iCBT proponents facilitate the implementation of iCBT [28]. However, fostering this motivation and changing the way clinicians practice is difficult [28,38,65]. One way to foster this motivation may be through routine monitoring of iCBT outcomes, which can provide persuasive evidence of intervention effectiveness to stakeholders as well as enhance its delivery. Services in Australia and Canada reported that they regularly conducted audits of service effectiveness [38,64,71], and such routine monitoring data were used so that staff could evaluate and understand the effectiveness of iCBT in their service [28,65] and to ensure compliance with treatment manual [64].

Third, effective marketing and service promotion are essential to spreading iCBT initiatives [9,28]. Advertisement campaigns (eg, web-based and print media) are frequently successful in recruiting participants for trials and routine care [52,55,64,66], though such marketing campaigns can require a large quantity of resources [28]. Recruitment and retention of therapists in iCBT-related positions is also critical. Clinician recruitment can be an issue as some therapists believe that iCBT limits professional freedom due to its highly structured working requirements and that the working conditions are not attractive [9,22].

Fourth, scaling of iCBT within services is challenging and requires consideration of multiple factors. The physical infrastructure (eg, internet) must be in place [49], funding needs to be procured [9], service decision makers must be convinced of the intervention’s feasibility [9], there needs to be evaluation frameworks for existing and new iCBT programs [22,37], and governance frameworks (eg, clinical, IT, and organizational) must be implemented that adhere to the wider legislative context [23]. Exploring new service pathways that are developed when considering iCBT services may allow for existing iCBT services to scale their offering [64]. A study stated that iCBT services should start with a small offering (eg, minimally monitored iCBT) and then acquire human and financial resources over time to build out the service [71].

##### Subdomain 2: The Practice of iCBT in the Workplace

###### Category 1: Appropriate Referral Pathways Are Necessary for the Delivery of iCBT

Reviewed articles suggested that successful implementation of iCBT requires the development of appropriate referral pathways, though there are many different referral strategies. Such pathways include self-referral [9,22,23,37,54,64], health care provider referral [22,23,38,40,46,52,54,56,61,62,64,66,70], access to pathways through marketing materials [52,55], contacting patients by email [63], contacting patients on waiting lists for face-to-face services [69], or clients applying through a secure website [71].

###### Category 2: Screening and Inclusion and Exclusion Criteria for iCBT Are Wide Ranging

Successfully implemented programs required patients to complete a web-based [22,23,28,37,54,63,64,66,68], in-person [23,48,61], or phone screening assessment [57,64,66]. As part of the screening, patients were asked about demographic information, mental health symptoms, commitment to iCBT, treatment history, level of risk, internet access, and language proficiency [28,37,45,48,61,63,64,66,68-71].

A variety of inclusion and exclusion criteria for accessing iCBT are reported as follows: no severe depression [37,59,63,71], no severe anxiety [63], no chronic or recurrent depression [47], no dementia [47], no past history of psychotic symptoms [28,64], aged >18 years [23,45,63,64,66,70,71], aged between 18 and 65 years [52], diagnosis of disorder via psychiatric interview or exceeding cutoff on established measure [23,37,45-47,50,52,64], no comorbid substance abuse [23,45,46,52,64,66] or use of benzodiazepines [46], no suicide risk [23,37,45,46,64,66,69-71], no bipolar disorder, psychosis, or obsessive compulsive disorder [23,45,46,52,66,69,71], adequate understanding of program language [45,46,50,52,66,71], no developmental disorders or other cognitive disabilities [69], no comorbidities or nonpsychiatric diseases that could cause depressive symptoms [70], no concurrent treatment [47,64,66,70,71], no change in medication before 1 month of commencing treatment [71], no email address or technological means to access treatment [46,82,70,71], patients with low motivation [37,64], and patients to far outside of the geographic location of the clinic [37,64,66,70].

###### Category 3: Use of, and Processes for, Providing Support That Are Crucial to the Provision of iCBT

Supported iCBT achieves positive clinical outcomes for patients [4,40,56-58], provides superior clinical and adherence outcomes over unguided interventions [4,40,56,64,67], and is regarded positively by the patients [41,69]. Therapeutic alliance is implicated as a mechanism behind the positive effects of supported interventions [48]; however, its effects are still unclear in iCBT, as it has been associated with positive outcomes [42] or no effect [48].

The purpose of support in iCBT is to “recognize and reinforce the participants’ work with the self-help material” [56] and promote engagement with the intervention [65,71]. The supporter in iCBT is posited to assume the role of a motivator, where the iCBT platform delivers the core treatment elements [38,56], and involves therapists monitoring patient progress [23,28,37,69], responding to their iCBT-related needs [28,67,69], and guiding the user through the initial setup [45,61]. Through written support, clinicians can encourage and affirm patients by expressing interest in the thoughts, feelings, and behaviors that patients share [50,57]. The quality of support affects client outcomes. For instance, leniency toward patient accountability (eg, homework completion) is associated with poorer patient outcomes [57]. Moreover, within written messages, the studies found that misspellings were frequent, emojis and emoticons were seldom used, and less detailed, shorter messages were associated with fewer web-based sessions completed [50].

iCBT support has been delivered in many ways [56]: in person [55,59], over email [38,64,71], by telephone [23,40,55,64,69,71], through the iCBT platform [23,45,50,55,63,66], through videoconferencing software [63], or by SMS text message [23]. Support can occur in real-time [58], on an “on-demand” basis [37], or asynchronously [48,58,64,66]. Support can be delivered weekly [23,47,55-57,61,64-66] or constantly through ongoing therapist monitoring [63]. Some programs incorporate homework assignments to inform clinicians when conducting support sessions [23,37]. In total, 3 programs implemented “step-wise” access to modules, where new content could not be accessed without completing a supported session [37], was unlocked 7 days after the completion of the previous module [69], or released gradually over an 8-week period [64].

Such decisions around how support is delivered and its frequency likely influence clinician time demand. Time spent delivering support varies widely [56], ranging from 10 to 100 minutes per session [23,38,40,44,55-57,59,61,64,69,71] and up to 8 hours per individual per course of treatment [38]. Support can be delivered weekly [23,47,55-57,64-66] or constantly through ongoing therapist monitoring [63]. Some programs involve 6 to 12 supporter telephone calls [40], emails from the supporter [52], or that the supporter contacts the patient ≥1 time a week for 8 weeks [47]. The end of treatment for some programs was based on a specific time or the number of support sessions received; for example, iCBT was cited across articles to be delivered over a varying course of 7 to 20 weeks [23,37,44,49,55,61,63,64].

###### Category 4: iCBT Can Be Successfully Implemented Across Supporters With a Variety of Personal and Professional Backgrounds

Successful implementation of iCBT included supporters who were volunteer peer-supporters with lived experience of the mental health condition [61], trained volunteers [49], psychologically trained experts (unspecified qualifications; [38]), clinical psychologists [9,23,47,50,61-64,66], psychiatrists [9], registered or provisionally registered mental health professionals [22,61], graduate students of psychology [22,66], trained health care professionals [49], psychologists in training [50,62], psychotherapists [62], social workers [62,64], mental health nurses [50], nurses [64], therapists with training on addictions [64], trained technicians [55,56,58], and general practitioners [59]. There is evidence that untrained technicians [56,58] or novice clinicians [57] achieve equal outcomes compared to trained clinicians, and that support from a technician is more effective than a waitlist control group [55].

##### Subdomain 3: Contextual Considerations for iCBT Implementation

###### Category 1: Governmental and Health Care Regulations Affect Implementation of iCBT

Governmental and health care regulations influence how iCBT can be implemented. An example of this is in Canada, where iCBT has been recognized by the Canadian government, which provides specific funding streams for iCBT services and research [28,64,65]. Other countries have implemented policies that incentivize the use of iCBT to improve access to psychological therapy [9,49]. Other regulations impacting iCBT include limitations placed on therapeutic contact taking place over the internet [48,49], requiring iCBT clinics to adhere to existing frameworks for the delivery of therapy [22,23,57] and policies around the delivery of therapy over the internet [57,62].

###### Category 2: Lack of Workforce Availability for iCBT as a Limiting Factor in the Provision of iCBT

The lack of workforce availability can limit the implementation of iCBT, particularly because as access to mental health care increases, so does the demand for services [38,42]. One study in Sweden observed only 1 to 2 therapists participating in iCBT initiatives among implementing services and further commented that due to face-to-face resources being expensive and scarce, a dedicated iCBT workforce could resolve this issue in terms of resource and cost [62]. Another study stated that the presence of trained iCBT professionals in certain health sectors (eg, veteran care in the United States) is rare [65].

###### Category 3: Cost Models and Cost-Effectiveness of iCBT Maybe an Asset for Successful Implementation

iCBT was provided to patients through the following 5 cost models: free of charge [59], through publicly funded health care systems [9,22,23,28,37,45,48], subsidized by health care providers [23,38], at a cost to patients when they are not within certain catchment areas or countries [38], or as part of insurance plans [48]. The establishment of reimbursement systems for iCBT was cited as an important factor for cost estimates in the future [9,48]. Moreover, it was hypothesized that as iCBT cost-effectiveness becomes more salient, health care providers (public or private) will advocate for it as a first-line intervention to efficiently gatekeep therapeutic resources [38]. iCBT did not incur extra costs to public health care systems [28] and was cost-effective (depending on the “willingness to pay” standards of the health care body) [70]. One study posited that a dedicated iCBT workforce should be developed to create a less expensive alternative [62].

## Discussion

### Principal Findings

Our mixed methods systematic review highlights the knowledge we have gained from the available literature on the implementation of iCBT. Some of our key findings regarding the process for implementing iCBT include the practice of iCBT, with special reference to determining client eligibility and effectively supporting patients in iCBT. The management of iCBT in the workplace, especially staff and operational considerations, also surfaced as an important process to consider when implementing. Other related findings include the importance of staff training, the management of treatment pathways, security, and factors for consideration within the wider context that impact the implementation of iCBT. In terms of implementation insights, this review has highlighted that clinician and patient attitudes toward iCBT can influence its ability to achieve intended outcomes and the need to continually tailor iCBT for patient benefit and that further research can help to develop our understanding of implementing iCBT successfully.

As would be expected, the practice of iCBT was highlighted as important to the implementation process for iCBT within a mental health service. For instance, what constitutes eligibility for an iCBT intervention manifested in two categories: (1) screening and inclusion criteria for accessing iCBT need to be thoroughly defined and (2) consideration of the usefulness of iCBT beyond the original target of mild-to-moderate depression and anxiety. Historically, eligibility for iCBT has been characterized by low symptom presence (mild to moderate) and no significant risk issues. This approach was sensible while establishing iCBT’s safety and effectiveness as an intervention, subsequently resulting in a well-validated evidence base supporting iCBT for treating depression and anxiety. Consequently, the preponderance of historical eligibility seems to be an artifact in need of revision. This is particularly important in light of the growing body of literature to support iCBT’s applicability to more severe presentations of mental health difficulties [72-75]. In addition, real-world data from iCBT clinics highlight that a substantial proportion of patients accessing these services have presentations within the moderate to severe range [22].

There is a clear need for services to consider the populations they serve (eg, general severity levels and client demographics) and tailor their model of iCBT provision. Still, despite the available evidence, clinical guidelines lag in their support for iCBT in extended service delivery pathways [76]. This situation poses some difficulty for certain services or health systems to innovate their use of digital interventions (eg, the improving access to psychological therapies program in England, which offers treatment based on National Institute for Health and Care Excellence guidelines). Specifically in the English context, the original guideline for the use of iCBT was rolled out in 2004 [77,78] and was updated in 2022. Since then, technologies and research have developed and would suggest the utility of iCBT for the broader population.

Our results highlight the importance of the operational aspects of iCBT services. First, the importance of effective management and leadership to support the implementation of iCBT was identified through the review. Transformational leadership approaches, that is, leadership styles associated with motivating and compelling employees to participate in a shared vision [79], have been found to be associated with increased levels of innovation climate, further defined as an organizational climate that is conducive to the adoption of novel, evidence-based practices [80]. Implementing iCBT requires leaders to navigate interactions across multiple levels of a service and motivate staff to ensure the vision of iCBT is fulfilled. However, the current studies identified do not illustrate in depth the effects of leadership nor was it their primary or secondary focus. Despite this, it is still important that this finding was communicated through this small pool of studies, and more research is needed to inform this gap in knowledge.

Training staff in iCBT and increasing their motivation to use it were both cited as important. As an in-service activity, training clinicians and therapists in the use of evidence-based practice has a substantial literature base [81,82]. However, our findings highlight variance in the training delivered to therapists charged with delivering iCBT, ranging from hours to up to a year of continued education, and the components of the training were also not described at length across articles. The wider literature on training stands in contrast to what we identified; training programs for evidence-based practice tend to produce better outcomes (eg, competency, evidence-based practice use, and positive attitudes) when multicomponent approaches are used (eg, workshop, follow-up, and audit of skills acquired) [81]. To date, no systematic evaluation of iCBT training programs has been conducted, and it has also been cited by one of the included studies that training programs for these interventions are rare [37]. Similarly, we identified that staff motivation to use iCBT needs to be fostered. This motivation can also be developed through training initiatives, where implementers can illustrate the benefits that iCBT brings to routine clinical practice (eg, improves patient symptoms and access to care, is usable, and is not time consuming), and this activity may potentially influence motivation around intervention use [83,84].

Furthermore, routine monitoring of the intervention and its outcomes was also cited as important for the continued development of iCBT within the service. This activity can allow supporters in iCBT to reflect on their own practice to improve service provision, with an article stating that clinicians who administer iCBT desire comprehensive updates regarding iCBT to understand its impact on wider service outcomes [28]. This activity is reminiscent of the construct “reflexive monitoring” from normalization process theory [84], where individual and group reflections on processes around a specific evidence-based practice can lead to revisions in practice that are adapted to best suit the needs and structures of the service context. The results regarding the operational aspects of iCBT, despite not being widely reported across the literature, indicate that factors associated with evidence-based practice success in the implementation literature are being considered when iCBT is implemented, which is a promising finding. More widespread reporting of this information could be beneficial to practicing professionals when making choices about using iCBT with their patients.

Patients tend to be positive about iCBT and the support they receive, but clinician attitudes generally lean toward the negative. From service illustrations, we can infer that clinicians receive significant exposure to iCBT when it is implemented [22,23,38], and its effectiveness is grounded in the literature. Negative attitudes can result in the abandonment of the implementation effort due to a lack of acceptance or misunderstandings around the perceived value of the treatment [85,86]. A better understanding of negative clinician attitudes can be attained if iCBT were to be interpreted as a novel, evidence-based practice. A literature search around clinicians’ attitudes toward evidence-based practice provides some insights, including that clinicians rate “other” sources of information (eg, colleague opinion and previous experience) as more impactful than published evidence on their decisions for treatment [87-89]. Fostering attitudes conducive to the uptake of evidence-based practice has been associated with transformational leadership styles [80] and systematic training initiatives that highlight how the evidence-based practice is integrated with the wider service system [82], both of which were evident within the current review. However, where there is a disconnect between clinicians and service management or staff does not understand the relative advantage (from the diffusion of innovations) [90], iCBT over existing practice can subsequently create barriers to evidence-based practice uptake [90,91]. This disconnect is well documented in implementation science theories, such as readiness for change [92] and implementation climate [93,94], both of which also emphasize the role of attitudes in evidence-based practice use and implementation.

iCBTs vary widely in their delivery (guided and self-guided), support time frames, and those who provide the support, but the take-home insight is that patients receive the interventions well in terms of satisfaction and clinical outcomes achieved. This malleability of iCBT, where it can assume many forms yet achieve the intended results, underlines the scalability of the intervention. A narrative review of factors associated with scaling public health interventions described that, once an intervention has proven its effectiveness in both small- and large-scale trials, management and practice factors, such as having systems for monitoring intervention performance, funding, and interacting with stakeholders within the wider health care system, become important for the scaling process [95]. Despite this, an “implementation gap” remains, where effectiveness reductions and high levels of attrition occur when we transition from efficacy settings to real-world service provision [96]. This further creates a treatment gap within users assigned iCBT as a treatment option, where implementation factors (eg, abandonment due to encountering bugs and not having a care provider to explore these with) may cause attrition, lowering the promise of scalability purported by these treatments [97].

### Limitations

In total, 4 main limitations were identified as part of this mixed methods systematic review. First, we used a targeted search strategy to produce a dataset that the authors acknowledge is incomplete due to a lack of proper use of terminology within the field to reference implementation. Therefore, we acknowledge that this review is not definitive on the implementation of iCBT and only reports on relevant factors within the articles identified. There is already a movement to standardize the reporting of digitally delivered psychological treatments in research studies (eg, use of CONSORT [Consolidated Standards of Reporting Trials] for eHealth) [98], and perhaps this should be succeeded by an attempt to standardize how we report implementation learnings too.

The second limitation consisted of the “blind spots” associated with the development of the analytic framework that may have resulted from the background of the researchers. The authors mainly come from a background in psychology, and none would consider themselves to be implementation specialists. Other review types (eg, realist, scoping, or narrative reviews) conducted by different research groups may uncover nuances that were otherwise unidentified by this review.

The third limitation of the study relates to the limitations present in the original papers included in this review. The heterogeneous nature of the articles included prevented a formal quality appraisal from being conducted. For instance, we had considered using a tool, such as the Critical Appraisal Skills Programme checklist, but this tool did not provide for a quality review of the narrative-type articles (eg, Schröder et al [56], Andersson et al [57], Andersson et al [58], and others) that were included. Furthermore, there are issues around assessing the quality of qualitative evidence within review-type studies and whether studies should be excluded based on perceived quality, which further compounds the issue [99-101]. Thus, we did not assess the quality of the included articles, and no articles were excluded based on methodological flaws. Relatedly, a few articles had a primary objective of exploring a facet of the implementation of iCBT for depression and anxiety, which is important to note when interpreting the results.

The fourth and final limitation of the study is whether an expansion of search terms could have been used. For instance, the term “computerized CBT” was not used in the search terms. However, while the search string did not include this term, we did include an abbreviated term for this, “ccbt,” and it is notable that by using this abbreviated term we identified specific studies that referenced computerized cognitive behavioral therapy (eg, Wells et al [44], Wright et al [4], Kenicer et al [49], Wright et al [51], and Grist and Cavanagh [54]). Regardless, it is possible that the search string may not have completely captured all possible studies.

### Conclusions

This mixed methods systematic review has identified several strategies for consideration when attempting to implement iCBT. Broadly, these strategies emphasize the importance of effective leadership, managing staff and operations associated with the practice of iCBT, implementing and developing professionals to provide the supported component of iCBT, accounting for context, and deriving implementation insights from novel research contributions in iCBT for patient benefit. Future research into iCBT in real-world settings should endeavor to supply appropriate supplemental information that details the efforts associated with implementing the intervention within care pathways. In tandem, efforts could be made to standardize practices that can support the transferability of learning and scalability through the use of a standardized lexicon of terms that are appropriately used.

## Data Availability

Data sharing is not applicable to this paper as no datasets were generated or analyzed during this study.

## Appendix

Multimedia Appendix 1 Search terms used within mixed methods systematic review.

Multimedia Appendix 2 Description of included articles within mixed methods systematic review.

Multimedia Appendix 3 References analyzed as part of this mixed methods systematic review.

Multimedia Appendix 4 PRISMA (Preferred Reporting Items for Systematic Reviews and Meta-Analyses) checklist.

## Abbreviations

CONSORT: Consolidated Standards of Reporting Trials
iCBT: internet-delivered cognitive behavioral therapy
PRISMA: Preferred Reporting Items for Systematic Reviews and Meta-Analyses
