# Supplementary material for: Implementing Internet-Delivered Cognitive Behavioral Therapy for Depression and Anxiety in Adults: Systematic Review
Source: J Med Internet Res. 2025 Jan 28;27:e47927. doi: 10.2196/47927 (PMC11815312; doi:10.2196/47927)
Supplement: Multimedia Appendix 1 [file jmir_v27i1e47927_app1.docx]

## **Appendix 1: Search terms used within mixed methods systematic review**


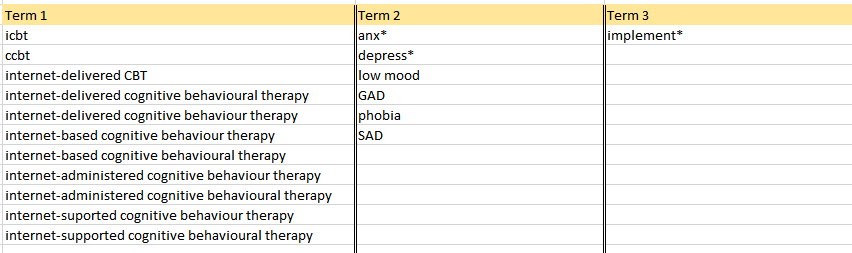


Search string 1 – EBSCO (PsycInfo, PsycArticles, Medline, CINAHL Complete)

**Term 1:** “ICBT” or “CCBT” or “internet-delivered CBT” or “internet-delivered cognitive behavioural therapy” or “internet-delivered cognitive behaviour therapy” or “internet-based cognitive behaviour therapy” or “internet-based cognitive behavioural therapy” or “internet-administered cognitive behaviour therapy” or “internet-administered cognitive behavioural therapy” or “internet-supported cognitive behaviour therapy” or “internet-supported cognitive behavioural therapy”

AND

**Term 2:** “Anx*” or “depress*” or “low mood” or “GAD” or “phobia” or “SAD”

AND

**Term 3:** “Implement*”

Search String 2 – EMBASE

(‘ICBT’ OR ‘CCBT’ OR ‘internet-delivered CBT’ OR ‘internet-delivered cognitive behavioural therapy’ OR ‘internet-delivered cognitive behaviour therapy’ OR ‘internet-based cognitive behaviour therapy’ OR ‘internet-based cognitive behavioural therapy’ OR ‘internet-administered cognitive behaviour therapy’ OR ‘internet-administered cognitive behavioural therapy’ OR ‘internet-supported cognitive behaviour therapy’ OR ‘internet-supported cognitive behavioural therapy’) AND (‘Anx*’ OR ‘depress*’ OR ‘low mood’ OR ‘GAD’ OR ‘phobia’ OR ‘SAD’) AND ‘Implement*’
