# Supplementary material for: Implementing Internet-Delivered Cognitive Behavioral Therapy for Depression and Anxiety in Adults: Systematic Review
Source: J Med Internet Res. 2025 Jan 28;27:e47927. doi: 10.2196/47927 (PMC11815312; doi:10.2196/47927)
Supplement: Multimedia Appendix 2 [file jmir_v27i1e47927_app2.docx]

## **Appendix 2 – Description of included papers within mixed methods systematic review**

| Paper ID | Paper type | Aims/Hypotheses/Objectives | Method Description | Results Summary |
| --- | --- | --- | --- | --- |
| 1 | Meta-analysis | Conduct an individual participant data meta-analysis to determine the prevalence of clinically significant deterioration in adults with depressive symptoms who received self-guided iCBT compared with control conditions | RCTs that reported results of self-guided iCBT compared with control conditions in adults with symptoms of depression | 13/16 eligible trials were included in the present IPD meta-analysis. 7.2% of participants showed clinically significant deterioration |
| 2 | Quantitative study | To evaluate the implementation of a third sector remote CCBT @Home eTherapy service for people experiencing common mental health problems supported by individuals with lived experience. | Supported CCBT packages with telephone support were delivered over a 30-month period. Self-complete measures identifying levels of depression, anxiety and functioning were administered at each treatment appointment. | 2/3 of all participants attended an initial assessment and 53.4% of referrals assigned to CCBT completed treatment. Statistically significant improvements in anxiety, depression and functioning were found |
| 3 | Mixed-methods study | Measuring acceptability, satisfaction, and efficacy of an iCBT program | self-reported online questionnares | Most respondents were satisfied with the programme (n = 191), felt supported (n = 203), reported positive gains and impact resulting from use of the programme, and perceived these to be likely to be lasting effects (n = 149 |
| 4 | Qualitative study | Identify the main implementation challenges perceived by therapists and managers involved in the practical operation of iCBT services in routine care settings in five European countries. | multiple comparative case study using interviews with management, focus group interviews, and demonstration of online programs | 1) integration in the mental health care system; 2) recruitment of patients; 3) working practice of therapists; and 4) long-term sustainability of service |
| 5 | Commentary | Discusses the strengths and limitations of internet-based cognitive-behavioral treatments (ICBT) for anxiety disorders | Analysis evaluating ICBT and comparing the course of treatment for patients with contrasting clinical outcomes, the authors offer insights into the many benefits and challenges of ICBT | Electronically-delivered interventions offer advantages, including increased access to treatment, a potential bridge to in-person therapy, and opportunities for large-scale delivery. ICBT can be improved, such as increased attention to patient motivation at the onset of treatment and specific strategies to enhance exposures, which we view as a critical ingredient to the treatment of anxiety disorders |
| 6 | Quantitative study | Investigate differences in icbt outcomes to more traditional in person therapy | Students completed online surveys | Less severe depression symptoms and female gender were predictors of higher ratings of ICBT acceptability. Students who had greater intentions to seek mental health services or were graduate students viewed ICBT as more credible than those who had lower intentions. |
| 7 | Meta-analysis | To examine evidence for the effectiveness of CCBT for depression in primary care and assess the impact of therapist supported CCBT vs self guided CCBT | searched for randomized studies of CCBT compared to control groups for treating depression in primary care settings. Meta analysis compared differences between post treatment mean scores in each condition as well as mean scores at follow up. | 8 studies met inclusion criteria. Effect size was g=0.258, indicating small but significant advantage for CCBT over control conditions. |
| 8 | Quantitative study | Evaluate the clinical effectiveness of iCBT for depression and anxiety in routine secondary care. | retrospective cohort study | large and significant reductions in the symptom levels of depression (beta=-6.27, SE 0.83, P<.001, d=1.0) and anxiety (beta=-3.78, SE 0.43, P<.001, d=1.1). High baseline severity of the primary disorder was associated with high treatment gains |
| 9 | Quantitative study | To evaluate the effect of iCBT on social anxiety disorder | Within group surveys and measures over a 3 year period | For social anxiety symptoms significant within-group effect sizes (post-treatment: d = 1.00–1.10; six-month follow-up: d = 1.03–1.55). Also significant effects on secondary depression symptoms (d = 0.67). Clinically significant improvement reported by 66.2% of the participants, and 16.6% a significant deterioration. |
| 10 | Meta-analysis | To evaluate the efficacy of computer-assisted forms of cognitive-behavior therapy for major depressive disorder and examine the role of clinician support and other factors that might affect outcomes. | 40 randomized controlled investigations of computer assisted cognitive behavior therapy for depression were included in meta-analysis. | overall mean effect sice for CCBT compated to control conditions was g=0.502, a moderately large effect. Completion rate and study setting also influenced outcomes. Self guided CCBT proved less effective then guided. |
| 11 | Quantitative study | Feasibility study for iCBT on depressed patients | pilot feasibility trial | clinically significant decrease (46%) in depressive syptoms |
| 12 | Case studies | To describe the setting, relationship to existing health services, procedures for referral, assessment, treatment, patients and outcomes of ICBT clinics in Sweden, Denmark, Norway, Canada and Australia. | A descriptive analysis of health clinics in 5 different countries | therapist-guided ICBT can be a valuable part of mental health services for anxiety and depression. Important components of successful ICBT services are rigorous governance to maintain a high standard of clinical care, and the measurement and reporting of outcomes. |
| 13 | Commentary | To review the evidence from effectiveness studies and highlight challenges when implementing ICBT. | commentary paper discussing iCBT in normal clinical settings | It's possible to transfer ICBT to clinical practice with sustained effects and moderate to large effect sizes. |
| 14 | Commentary | To provide takeaways learned from successful digital mental health services | commentary paper about lessons learned from establishing and delivering iCBT methods | DMHS should provide not only treatment but also information and assessment services, that DMHS require robust systems for training and supervising therapists, that specialist skills are required to operate DMHS, and that the outcome data from DMHS can inform future mental health policy |
| 15 | Quantitative study | Investigate Scottish health service infrastructure and policies that promote or impede the implementation of cCBT in the NHS | national survey | Reported need for software for iCBT use, lack of computer available for patient use, and obstructive local policy |
| 16 | Mixed-methods study | Evaluate therapist feedback (written reviews) sent during iCBT provision | content analysis to identify therapist behaviours | most frequently used therapist behaviours were informing, encouraging, and affirming. However, these were not related to patient outcomes. Although infrequently used, confronting was positively correlated with session completion (ρ=.342, P=.02). |
| 17 | Systematic review | To review research on computer-assisted cognitive-behavior therapy (CCBT) performed in medical settings with the goals of assessing the effectiveness of this newer method of treatment delivery, evaluating the need for clinician support of therapeutic computer programs, and making suggestions for future research and clinical implementation. | systematic review of randomized controlled trials | CCBT can be an effective treatment for depression in primary care patients and health care anxiety. Also, it can be a useful component of treatment for somatic conditions including irritable bowel syndrome, diabetes, fibromyalgia, and chronic pain |
| 18 | Quantitative study | To examine whether attitudes toward Internet interventions moderate the effects of a depression-focused Internet intervention, and how attitudes change over the course of treatment among those who do or do not benefit. | Subgroup analysis of the randomized controlled EVIDENT trial | Positive initial attitudes toward Internet interventions were associated with greater efficacy independent of usage time, whereas a negative attitude was associated with reduced efficacy |
| 19 | Mixed-methods | To review client's emails to gather insights about negative effects from iCBT | directed content analysis to examine emails for mentions of negative affects. Correlational analysis conducted between negative effects and 5 additional measures | over half of participants evaluated mentioned at least one negative experience from iCBT |
| 20 | Meta-analysis | To evaluate the effectiveness of computerised cognitive behavioural therapy (CCBT) as a low intensity intervention for common mental health disorders (CMHD), and investigates some potential moderators of these effects | A meta- analysis was conducted on 49 randomised controlled trials comparing CCBT to other therapies (n = 24) and waiting list controls (n = 25), across the range of CMHD | an overall mean effect size of g = 0.77 (95 % CI 0.59–0.95) in favour of the CCBT trial arms. CCBT was found to be significantly more effective than both waitlist and active control conditions. |
| 21 | Systematic review | To evaluate whether internet-delivered psychological treatments for mood and anxiety disorders are efficacious, noninferior to established treatments, safe, and cost-effective for children, adolescents and adults | 52 relevant RCTs were identified whereof 12 were excluded due to high risk of bias. Trials evaluated internet-delivered cognitive behavioral therapy (I-CBT) against a waiting list in adult volunteers | iCBT is a viable treatment option for adults with depression and some anxiety disorders who request this treatment modality |
| 22 | Quantitative study | To evaluate the effectiveness of ICBT in the treatment of social anxiety disorder and to determine the significance of patient adherence and the clinic’s years of experience in delivering ICBT | A longitudinal cohort study where were patients treated with ICBT at an outpatient psychiatric clinic. Primary outcome measure was the Liebowitz Social Anxiety Scale–Self-Rated | Reduction in rates of social anxiety after treatment. These improvements were sustained at the 6-month follow up. Positive association between clinic's experience with CBT and observed treatment outcome. |
| 23 | Review | Re-investigate evidence into effectiveness of interventions for depressive symptoms | Review of internet intervention evidence | There is a strong need for mental health care at a low threshold. ICBT can fill this roll, either guided or self-guided |
| 24 | Review | To review the treatment approach and the evidence base, arguing that ICBT can be viewed as a vehicle for innovation | Review of studies testing ICBT and CBT practices. Studies on the possible harmful effects of ICBT are also reviewed. | ICBT and other forms of Internet interventions hold promise as a way to increase access to evidence based psychological treatment. They can also serve as vehicles for innovation, which may subsequently inform face­to­face treatments. |
| 25 | Commentary | To review previous research regarding methods of implementing iCBT | Commentary discussing the current research on iCBT delivery mwthods | Use of the internet for delivering CBT has been found to be effective in several randomized controlled trials and programs should consider the importance of proper patient diagnoses, evaluation of suitability and user friendliness of the internet system |
| 26 | Qualitative study | To explore aspects perceived by GPs to affect the implementation of guided ICBT in daily practice. Understanding their perspectives may contribute to improving the treatment of depression in the context of general practice. | A training package introducing a Norwegian translation of the ICBT program MoodGYM was developed and presented to GPs in Norway. Following training, GPs were asked to include guided ICBT in their regular care of patients with symptoms of depression by providing brief, face-to-face follow-up consultations between modules. We interviewed 11 GPs who had taken the course | ICBT motivated them to invest time and effort in improving treatment. The most important motivating aspects in MoodGYM were that a program based on cognitive behavioral therapy could add a structured agenda to their consultations and empower depressed patients. |
| 27 | Review | To review the research evidence with reference to efficacy and effectiveness and presenting a model for dissem- ination and uptake of iCBT into practice | Review includes studies of participants who would meet criteria for major depressive disorder who were supported as they learnt and implemented changes in thoughts, emotions and behaviours by using cognitive behaviour principles | This form of treatment is effective and acceptable to both patients and clinicians. |
| 28 | Meta-analysis | Meta-analysis of CCBT studies for depression | Meta-analysis of 14 trials | For the sixteen comparisons (2807 participants) comparing CCBT and control conditions, the pooled SMD was −0.48 [95% IC −0.63 to −0.33], suggesting similar effect to the past reviews. Also, there was no significant clinical effect at long follow-up and no improvement of function found. Furthermore, a significantly higher drop-out rate was found for CCBT than for controls. |
| 29 | Qualitative study | Conduct a parallel process evaluation designed to understand facilitators and barriers impacting the uptake and implementation of ICBT. | Process evaluation - therapists and managers completed online surveys | ICBT implementation was perceived prominently facilitated by intervention characteristics and implementation processes |
| 30 | Quantitative study | To test the generalizability of this finding to the implementation of CCBT in a service user-led, third sector Self Help Clinic. | 510 referrals for the Beating the Blues program were received over a 16 month period in routine care. The PHQ-9 and GAD-7 Scales were administered pre-treatment and during each treatment session. The CORE-OM, Work and Social Adjustment Scale and Patient Experience Questionnaire were also administered pre-treatment and immediately on completing treatment | More than two-thirds of referrals were suitable for treatment and completed a baseline assessment; 84% of these started the Beating the Blues program. CCBT can be effectively implemented in a service user-led, third sector Self Help Clinic, increasing access to psychological therapies to meet local needs for tier two interventions for depression and anxiety |
| 31 | Quantitative study | To identify and describe primary care organizations providing ICBT in Sweden and compare decision makers’ views on barriers and facilitators to implementation of ICBT among ICBT implementers and non-implementers | An online survey based on a checklist for identifying barriers and facilitators to implementation was distributed to participants | 89.8% of the participating organizations provided CBT. 20.5% of organizations offered ICBT. Most professionals delivering ICBT were psychologists (80%) and social workers (37%). The majority (73%) of organizations had 1 to 2 persons delivering ICBT interventions. |
| 32 | Randomized controlled trial | To assess the implementation of a highly structured therapist-guided iCBT programme for people with work-related anxiety and depression, in terms of programme efficacy, participants' adherence and satisfaction | 12 videoconference sessions that took place across 17–20 weeks. | All participants endorsed lower depression (BDI-II F(1) = 36.98, p < .001; ATQ F(1) = 24.22,  p < .001), and anxiety (STAI-State F(1) = 76.62, p < .001) after the programme. |
| 33 | Quantitative study | To examine trends in utilization, patient characteristics, and longitudinal improvements for patients receiving transdiagnostic iCBT | Patients engaged in telephone screenings where demographics and mental health history was collected and completed measures at pre-treatment, post-treatment and at 3- to 4-month follow-up | Primary reason for referral to another service was high suicide risk/severe symptoms (47.1%). Examination of trends showed growing use of transdiagnostic iCBT over time (37% increase per year). There was remarkable stability in patient characteristics across years. Significant longitudinal improvements observed. |
| 34 | Qualitative study | To examine PC-MHI mental health clinicians’  perspectives on adapting collaborative care models to support  cCBT for VA primary care patients. | Carried out structured interviews with PC-MHI nurse care managers,  licensed social workers, psychologists, and psychiatrists in  one VA health-care system | cCBT awareness and knowledge were not widespread, but participants were still  highly accepting of enhancing PC-MHI models with cCBT for depression treatment |
| 35 | Pilot randomized controlled trial | To examine the efficacy of an internet-delivered cognitive behavioural treatment (ICBT) in an Arabic-speaking immigrant population | Pilot study in which interventions consisted of nine modules targeting areas such as depression, anxiety and insomnia. Self-reported symptoms of depression on the PHQ-9 were used as primary outcome measure. Secondary outcome measures of anxiety, stress, insomnia, quality of life and post-traumatic stress were also used | depressive symptoms were significantly reduced compared to the wait-list control group with a between group effect at post-treatment of Cohen’s d = 0.85 [0.29, 1.41]. |
| 36 | Systematic review | Produce a critical appraisal of published reviews about the acceptability of cCBT for adults | Umbrella review; synthesize quantitative findings relating to acceptability of and adherence to cCBT for common adult mental disorders | review indicated that “one size did not fit all” regarding the acceptability of cCBT and that individual tailoring of cCBT is required in order to increase population reach, uptake, and adherence and therefore, deliver treatment benefits and improve mental health. |
| 37 | Quantitative study | To evaluate perceptions around iCBT | Online surveys distributed to participants | No differences in perceptions of ICBT were identified between the conditions. Ratings of credibility, treatment expectancy, anticipated treatment adherence, and acceptability suggested that PSP had positive perceptions of ICBT |
| 38 | Qualitative study | To evaluate patients who had undergone iCBT with minimal support while actively awaiting outpatient psychological treatment in the form of face-to-face CBT | Semi-structured interviews | iCBT treatment was unfavorably compared to the usual face-to-face treatment at the clinic. |
| 39 | Quantitative study | To assess the cost-effectiveness of implementing a community internet-based cognitive behavioral therapy intervention for treating major depressive disorder. | Cost data collected from participants using the program. The health states, transition probabilities, and utilities were computed using Patient Health Questionnaire–9 scores. | intervention was more costly than usual care; the discounted (3%) and non discounted incremental cost-effectiveness ratios were €29,367 and €26,484 per quality-adjusted life-year, respectively (approximately US $35,299 and $31,833, respectively). |
| 40 | Randomized controlled trial | To evaluate the efficacy of an iCBT transdiagnostic program translated from English to French and offered in Canada using a minimally monitored delivery model for the treatment of anxiety and depression | RCT using GAD-7 and PHQ-9 as primary outcome measures | treatment group had significantly lower PHQ-9 and GAD-7 scores post-treatment than controls with small between-groups effect sizes (d = 0.34 and 0.37) |
