# Supplementary material for: Implementing Internet-Delivered Cognitive Behavioral Therapy for Depression and Anxiety in Adults: Systematic Review
Source: J Med Internet Res. 2025 Jan 28;27:e47927. doi: 10.2196/47927 (PMC11815312; doi:10.2196/47927)
Supplement: Multimedia Appendix 3 [file jmir_v27i1e47927_app3.docx]

**Appendix 3. References analysed as part of mixed methods systematic review**

| **Reference** |
| --- |
| 1. Karyotaki, E., Kemmeren, L., Riper, H., Twisk, J., Hoogendoorn, A., Kleiboer, A., … & Cuijpers, P. (2018). Is self-guided internet-based cognitive behavioural therapy (iCBT) harmful? An individual participant data meta-analysis. Psychological medicine, 48(15), 2456-2466. |
| 2. Gellatly, J., Chisnall, L., Seccombe, N., Ragan, K., Lidbetter, N., & Cavanagh, K. (2018). @ Home etherapy service for people with common mental health problems: an evaluation. Behavioural and cognitive psychotherapy, 46(1), 115-120. |
| 3. Richards, D., Murphy, T., Viganó, N., Timulak, L., Doherty, G., Sharry, J., & Hayes, C. (2016). Acceptability, satisfaction and perceived efficacy of “Space from Depression” an internet-delivered treatment for depression. Internet Interventions, 5, 12-22. |
| 4. Folker, A. P., Mathiasen, K., Lauridsen, S. M., Stenderup, E., Dozeman, E., & Folker, M. P. (2018). Implementing internet-delivered cognitive behavior therapy for common mental health disorders: A comparative case study of implementation challenges perceived by therapists and managers in five European internet services. Internet Interventions, 11, 60-70. |
| 5. Woods, A. P., Stults, C. B., Terry, R. L., & Rego, S. A. (2017). Strengths and limitations of internet-based cognitive-behavioral treatments for anxiety disorders. Pragmatic Case Studies in Psychotherapy, 13(3), 271-283. |
| 6. Peynenburg, V. A., Mehta, S., & Hadjistavropoulos, H. D. (2020). Postsecondary student perceptions and preferences for the treatment of depression and anxiety: Comparison of internet-delivered cognitive behaviour therapy to face-to-face cognitive behaviour therapy and medication. Canadian Journal of Behavioural Science/Revue canadienne des sciences du comportement, 52(3), 220. |
| 7. Wells, M. J., Owen, J. J., McCray, L. W., Bishop, L. B., Eells, T. D., Brown, G. K., ... & Wright, J. H. (2018). Computer-assisted cognitive-behavior therapy for depression in primary care: systematic review and meta-analysis. *The primary care companion for CNS disorders*, *20*(2), 0-0. |
| 8. Mathiasen, K., Riper, H., Andersen, T. E., & Roessler, K. K. (2018). Guided internet-based cognitive behavioral therapy for adult depression and anxiety in routine secondary care: observational study. Journal of medical Internet research, 20(11), e10927. |
| 9. Nordgreen, T., Gjestad, R., Andersson, G., Carlbring, P., & Havik, O. E. (2018). The effectiveness of guided internet-based cognitive behavioral therapy for social anxiety disorder in a routine care setting. Internet interventions, 13, 24-29. |
| 10. Wright, J. H., Owen, J. J., Richards, D., Eells, T. D., Richardson, T., Brown, G. K., ... & Thase, M. E. (2019). Computer-assisted cognitive-behavior therapy for depression: a systematic review and meta-analysis. The Journal of clinical psychiatry, 80(2), 0-0. |
| 11. Whiteside, U., Richards, J., Bradley Steinfeld, G. S., Caka, S., Tachibana, C., Stuckey, S., & Ludman, E. (2014). Online cognitive behavioral therapy for depressed primary care patients: a pilot feasibility project. The Permanente Journal, 18(2), 21. |
| 12. Titov, N., Dear, B., Nielssen, O., Staples, L., Hadjistavropoulos, H., Nugent, M., ... & Kaldo, V. (2018). ICBT in routine care: a descriptive analysis of successful clinics in five countries. Internet interventions, 13, 108-115. |
| 13. Andersson, G., & Hedman, E. (2013). Effectiveness of guided internet-based cognitive behavior therapy in regular clinical settings. Verhaltenstherapie, 23(3), 140-148. |
| 14.Titov, N., Hadjistavropoulos, H. D., Nielssen, O., Mohr, D. C., Andersson, G., & Dear, B. F. (2019). From research to practice: ten lessons in delivering digital mental health services. Journal of clinical medicine, 8(8), 1239. |
| 15. Kenicer, D., McClay, C. A., & Williams, C. (2012). A national survey of health service infrastructure and policy impacts on access to computerised CBT in Scotland. BMC Medical Informatics and Decision Making, 12(1), 1-5. |
| 16. Mol, M., Dozeman, E., Provoost, S., Van Schaik, A., Riper, H., & Smit, J. H. (2018). Behind the scenes of online therapeutic feedback in blended therapy for depression: mixed-methods observational study. Journal of medical Internet research, 20(5), e9890. |
| 17. Wright, J. H., McCray, L. W., Eells, T. D., Gopalraj, R., & Bishop, L. B. (2018). Computer-assisted cognitive-behavior therapy in medical care settings. Current psychiatry reports, 20(10), 1-9. |
| 18. Schröder, J., Berger, T., Meyer, B., Lutz, W., Späth, C., Michel, P., ... & Moritz, S. (2018). Impact and change of attitudes toward Internet interventions within a randomized controlled trial on individuals with depression symptoms. Depression and anxiety, 35(5), 421-430. |
| 19. Gullickson, K. M., Hadjistavropoulos, H. D., Dear, B. F., & Titov, N. (2019). Negative effects associated with internet-delivered cognitive behaviour therapy: an analysis of client emails. Internet interventions, 18, 100278. |
| 20. Grist, R., & Cavanagh, K. (2013). Computerised cognitive behavioural therapy for common mental health disorders, what works, for whom under what circumstances? A systematic review and meta-analysis. Journal of Contemporary Psychotherapy, 43(4), 243-251. |
| 21. Arnberg, F. K., Linton, S. J., Hultcrantz, M., Heintz, E., & Jonsson, U. (2014). Internet-delivered psychological treatments for mood and anxiety disorders: a systematic review of their efficacy, safety, and cost-effectiveness. PloS one, 9(5), e98118. |
| 22. El Alaoui, S., Hedman, E., Kaldo, V., Hesser, H., Kraepelien, M., Andersson, E., ... & Lindefors, N. (2015). Effectiveness of Internet-based cognitive–behavior therapy for social anxiety disorder in clinical psychiatry. Journal of consulting and clinical psychology, 83(5), 902. |
| 23. Schröder, J., Berger, T., Westermann, S., Klein, J. P., & Moritz, S. (2016). Internet interventions for depression: new developments. Dialogues in clinical neuroscience, 18(2), 203. |
| 24. Andersson, G., Titov, N., Dear, B. F., Rozental, A., & Carlbring, P. (2019). Internet‐delivered psychological treatments: from innovation to implementation. World Psychiatry, 18(1), 20-28. |
| 25. Andersson, G. (2010). The promise and pitfalls of the internet for cognitive behavioral therapy. *BMC medicine*, *8*(1), 1-5. |
| 26. Wilhelmsen, M., Høifødt, R. S., Kolstrup, N., Eisemann, M., Chenhall, R., & Risør, M. B. (2014). Norwegian general practitioners’ perspectives on implementation of a guided web-based cognitive behavioral therapy for depression: a qualitative study. Journal of medical Internet research, 16(9), e208. |
| 27. Andrews, G., & Williams, A. D. (2015). Up-scaling clinician assisted internet cognitive behavioural therapy (iCBT) for depression: a model for dissemination into primary care. Clinical psychology review, 41, 40-48. |
| 28. So, M., Yamaguchi, S., Hashimoto, S., Sado, M., Furukawa, T. A., & McCrone, P. (2013). Is computerised CBT really helpful for adult depression?-A meta-analytic re-evaluation of CCBT for adult depression in terms of clinical implementation and methodological validity. BMC psychiatry, 13(1), 1-14. |
| 29. Hadjistavropoulos, H. D., Nugent, M. M., Dirkse, D., & Pugh, N. (2017). Implementation of internet-delivered cognitive behavior therapy within community mental health clinics: a process evaluation using the consolidated framework for implementation research. BMC psychiatry, 17(1), 1-15. |
| 30. Cavanagh, K., Seccombe, N., & Lidbetter, N. (2011). The implementation of computerized cognitive behavioural therapies in a service user-led, third sector self help clinic. Behavioural and Cognitive Psychotherapy, 39(4), 427-442. |
| 31. Brantnell, A., Woodford, J., Baraldi, E., van Achterberg, T., & von Essen, L. (2020). Views of Implementers and Nonimplementers of Internet-Administered Cognitive Behavioral Therapy for Depression and Anxiety: Survey of Primary Care Decision Makers in Sweden. *Journal of medical Internet research*, *22*(8), e18033. |
| 32. Elvira, A. C., Ivars, M. S., Giráldez, C. M., & Shih, P. C. (2021). Internet-based cognitive behavioural therapy programme with and without videoconference guidance sessions: A randomized controlled trial to treat work-related symptoms of anxiety and depression. *Clinical psychology & psychotherapy*, *28*(5), 1230-1242. |
| 33. Hadjistavropoulos, H. D., Peynenburg, V., Thiessen, D. L., Nugent, M., Karin, E., Staples, L., ... & Titov, N. (2021). Utilization, Patient Characteristics, and Longitudinal Improvements among Patients from a Provincially Funded Transdiagnostic Internet-delivered Cognitive Behavioural Therapy Program: Observational Study of Trends over 6 Years. The Canadian Journal of Psychiatry, 07067437211006873. |
| 34. Leung, L. B., Dyer, K. E., Yano, E. M., Young, A. S., Rubenstein, L. V., & Hamilton, A. B. (2020). Collaborative care clinician perceptions of computerized cognitive behavioral therapy for depression in primary care. Translational behavioral medicine, 10(3), 565-572. |
| 35. Lindegaard, T., Seaton, F., Halaj, A., Berg, M., Kashoush, F., Barchini, R., ... & Andersson, G. (2021). Internet-based cognitive behavioural therapy for depression and anxiety among Arabic-speaking individuals in Sweden: a pilot randomized controlled trial. Cognitive Behaviour Therapy, 50(1), 47-66. |
| 36. Treanor, C. J., Kouvonen, A., Lallukka, T., & Donnelly, M. (2021). Acceptability of Computerized Cognitive Behavioral Therapy for Adults: Umbrella Review. JMIR mental health, 8(7), e23091. |
| 37. McCall, H. C., Sison, A. P., Burnett, J. L., Beahm, J. D., & Hadjistavropoulos, H. D. (2020). Exploring perceptions of internet-delivered cognitive behaviour therapy among public safety personnel: informing dissemination efforts. International Journal of Environmental Research and Public Health, 17(17), 6026. |
| 38. Pedersen, M. K., Mohammadi, R., Mathiasen, K., & Elmose, M. (2020). Internet‐based cognitive behavioral therapy for anxiety in an outpatient specialized care setting: A qualitative study of the patients’ experience of the therapy. Scandinavian Journal of Psychology, 61(6), 846-854. |
| 39. Piera-Jiménez, J., Etzelmueller, A., Kolovos, S., Folkvord, F., & Lupiáñez-Villanueva, F. (2021). Guided Internet-Based Cognitive Behavioral Therapy for Depression: Implementation Cost-Effectiveness Study. Journal of medical Internet research, 23(5), e27410. |
| 40. Robichaud, M., Talbot, F., Titov, N., Dear, B. F., Hadjistavropoulos, H. D., Hadjistavropoulos, T., & Jbilou, J. (2020). Facilitating access to iCBT: a randomized controlled trial assessing a translated version of an empirically validated program using a minimally monitored delivery model. Behavioural and cognitive psychotherapy, 48(2), 185-202. |
